# The Management of Postoperative Recurrence in Crohn’s Disease

**DOI:** 10.3390/jcm13010119

**Published:** 2023-12-25

**Authors:** Ernesto Fasulo, Ferdinando D’Amico, Laura Osorio, Mariangela Allocca, Gionata Fiorino, Alessandra Zilli, Tommaso Lorenzo Parigi, Silvio Danese, Federica Furfaro

**Affiliations:** 1 Department of Gastroenterology and Gastrointestinal Endoscopy, IRCCS San Raffaele Scientific Institute, 20132 Milan, Italy; fasulo.ernesto@hsr.it (E.F.); damico.ferdinando@hsr.it (F.D.); allocca.mariangela@hsr.it (M.A.); fiorino.gionata@hsr.it (G.F.); zilli.alessandra@hsr.it (A.Z.); danese.silvio@hsr.it (S.D.); 2 Department of Biomedical Sciences, Humanitas University, Pieve Emanuele, 20089 Milan, Italy; 3 Gastroenterologist Hospital Pablo Tobon Uribe, Medellín 050010, Colombia; losorio@hptu.org.co; 4 Gastroenterology and Gastrointestinal Endoscopy, Vita-Salute San Raffaele University, 20132 Milan, Italy

**Keywords:** Crohn’s disease, ileocecal resection, postoperative, recurrence

## Abstract

Crohn’s disease (CD) is a chronic inflammatory bowel disease with different phenotypes of presentation, inflammatory, penetrating, or stricturing disease, that significantly impacts patient well-being and quality of life. Despite advances in medical therapy, surgery sometimes represents the only treatment to address complications, such as strictures, fistulas, or abscesses. Minimizing postoperative recurrence (POR) remains a major challenge for both clinicians and patients; consequently, various therapeutic strategies have been developed to prevent or delay POR. The current review outlines an updated overview of POR management. We focused on diagnostic assessment, which included endoscopic examination, biochemical analyses, and cross-sectional imaging techniques, all crucial tools used to accurately diagnose this condition. Additionally, we delved into the associated risk factors contributing to POR development. Furthermore, we examined recent advances in the prophylaxis and treatment of POR in CD.

## 1. Introduction

Crohn’s disease (CD) is a chronic immune-mediated inflammatory disorder characterized by intermittent periods of symptom exacerbation and inflammatory activity, affecting patients’ quality of life [1,2,3]. Medical therapy has advanced significantly in recent years, but often surgery remains necessary [4]. Half of the patients present with complications such as penetrating and stricturing disease, and at least 30–50% of patients undergo surgery in the first decade after diagnosis [5,6,7]. Unfortunately, surgery is not curative, and 35% of patients require a second surgical resection within 10 years of the first intervention [8]. In recent years, there has been a trend toward a decrease in ileocecal resection (ICR) [9,10], as reported in a national registry, that has shown a reduction from 36.8% in 1990–1995 to 12.4% in 2009–2014 [11].

Moreover, the introduction of biological agents has reshaped the therapeutic landscape for CD, leading to a reduction of recurrence rates in operated patients [12].

The POCER trial marked the turning point in the clinical management of postoperative recurrence (POR) in CD [13]. By introducing a personalized treatment approach based on individual risk factors, incorporating early colonoscopies, and targeted therapy, its findings paved the way for more effective strategies in these specific clinical scenarios. It also sparked significant research interest, leading to numerous studies that have explored this condition, thus bringing forth important advancements.

This review aims to summarize recent advancements in identifying risk factors for POR in CD, as well as the utilization of biomarkers, imaging techniques and endoscopy for monitoring, diagnosing, and managing recurrence. Additionally, emerging medical therapeutic approaches are discussed. 

## 2. Methods

A comprehensive literature search was conducted using PubMed, Medline, and Embase to identify all English-language articles published up to September 2023 that evaluate the management of postoperative recurrence in CD. The search was conducted using a combination of keywords, such as “Crohn”, “Crohn’s disease”, “CD”, “postoperative”, “recurrence”, “POR”, “risk factors”, “ileocecal resection”, “ICR”, “surgery”, “therapy”, “treatment”, and “prophylaxis”.

The reference lists of relevant abstracts, original articles, and reviews were reviewed, but only articles published in peer-reviewed journals were included, with a preference given to the most recent studies.

## 3. Monitoring

The diagnosis of postoperative recurrence (POR) of CD can involve multiple factors, such as clinical symptoms, serum and fecal markers, radiological assessment, and endoscopic findings [14]. However, distinguishing POR symptoms from other postoperative conditions, like pain resulting from adhesional obstruction, calculi, dysmotility, or diarrhea caused by bile-salt malabsorption or bacterial overgrowth, can be challenging [15]. Consequently, it is crucial to monitor patients to detect recurrence at an early stage and ideally implement preventive measures.

### 3.1. Endoscopy

Endoscopic evaluation is the gold standard for diagnosing POR [16]. For the assessment of endoscopic recurrence, the Rutgeerts’ score (RS) has been widely adopted in clinical practice, and it is routinely used in clinical trials (Table 1) [17]. In 1990, Rutgeerts et al. conducted a study demonstrating that the severity of endoscopic lesions observed within one year after surgery at the neoterminal ileum and ileocolonic anastomosis predict the likelihood of clinical activity in the future. This research revealed that 73% and 85% of patients had endoscopic lesions at 1- and 3-years following surgery, respectively [18]. The severity of endoscopic lesions in the RS is categorized into five levels: i0 no lesions; i1 up to five aphthous lesions in the neoterminal ileum; i2 more than five aphthous lesions in the neoterminal ileum with normal mucosa between the lesions, or skip areas of larger lesions, or lesion confined to the ileocolonic anastomosis (i.e., less than one centimeter in length); i3 diffuse aphthous ileitis with diffusely inflamed mucosa; and i4 diffuse inflammation of the neoterminal ileum with already larger ulcers, nodules and/or narrowing [18]. The definition of endoscopic recurrence is RS ≥ i2 because the recurrence of symptoms was seen in less than 20% of cases with i0–i1 [18]. A prospective study was conducted on CD patients who underwent an ileocolonoscopy six months after ICR, followed by clinical monitoring for a period of five years. The results of this study further supported the previously mentioned findings [19]. Clinical recurrence occurred in 11% (95% CI −1–23) of patients with i0–i1 compared to 57% (95% CI 20–94) with an i2 score, 75% of patients with an i3 score, and 100% with an i4 score (*p* = 0.001), showing a positive correlation between the endoscopic severity of the proximal site of the anastomosis at six months after surgery and the clinical recurrence rate during the following five years. Recently, a systematic review and meta-analysis by Ble et al. found that 33.4% of patients who experienced endoscopic recurrence—defined as RS ≥ i2—also faced clinical recurrence— defined according to the definition in each study, with a relative risk (RR) of 10.7 (95% CI 4.08–28.4) [20].

Even though RS is widely used, there are some limitations to be mentioned. The score has not been formally validated, and the interobserver reproducibility is moderate, especially when differentiating <i2 from ≥i2, which can lead to incorrect decisions in at least 10% of patients [21]. Moreover, the anastomotic lesions portend a better prognosis compared to neoterminal ileum lesions; therefore, a modified RS score was developed, which separates i2. Isolated lesions confined to the anastomosis are i2a, and the remaining lesions included in the i2 stage (>5 aphthous ulcers or larger areas of skip lesions) are classified as i2b [22,23,24]. In recent years, several papers have been published related to the different outcomes of patients with i2a vs. i2b lesions [25,26,27] without consistent findings; consequently, it is still unclear whether i2a and i2b findings should be treated and monitored differently. Recent advances in surgical approaches and different anastomotic techniques have highlighted the importance of considering various operative factors and led to the development of different surgical options, each with a different risk of POR [28,29,30]. Evidence indicates stapled intestinal anastomoses, whether side-to-side or end-to-end, demonstrate superior outcomes relative to hand-sewn techniques (anastomotic leak rate of 2% vs. 14%) [31,32]. Additionally, a systematic review and meta-analysis compared endoscopic POR rates in CD patients based on the ileocolonic anastomosis technique, specifically the conventional types (side-to-side, end-to-end, or end-to-side) or Kono-S anastomosis [33]. Seventeen studies that included 2087 patients who underwent surgical resection were analyzed. Of these, 17.7% of patients received Kono-S, while 81% had conventional anastomosis. The overall endoscopic POR rate was 37.2% (95% CI 27.7–47.2, *p* < 0.0001). In particular, the recurrence rate was lower following a Kono-S anastomosis, at 24.7% (95% CI 6.8–49.4), compared to 42.6% for conventional anastomoses (95% CI 32.2–53.4) [33].

In this constantly evolving context, developing a risk stratification scoring system that encompasses these differences and possesses valid predictive ability is essential [34]. Riviere et al. proposed a new recurrence score accounting for several anatomic locations with revised terminology for endoscopic POR [35]. Specifically, it differentiates lesions based on their location, proximal or distal to the ileal inlet, which is defined as the entry point of the neoterminal ileum into the anastomosis. The definition of POR corresponds to i2b or higher lesions in the neoterminal ileum involving the ileal inlet or body (segment of ileum between the anastomotic line and the ileal inlet), excluding other defined areas [35].

However, the timing of performing an ileocolonoscopy is crucial. If conducted too early after surgery, it may not detect patients who are likely to develop recurrent disease. On the other hand, if performed too late, the disease may have already become well-established [36]. An optimal time for an ileocolonoscopy assessment at six months was supported by the POCER trial, where 174 CD patients who underwent ICR received a 3-month metronidazole therapy and patients at high risk of recurrence (smokers, penetrating disease, and previous resection) also received thiopurine or adalimumab if intolerant to thiopurines. Patients were randomized either to active care with an ileocolonoscopy performed at six months after surgery and stepped-up therapy if an endoscopic recurrence occurred (RS ≥ i2) or to no endoscopy group. Endoscopic recurrence at 18 months was registered in 67% of patients in the standard care group in contrast to 49% in the active care group (*p* = 0.03) [13]. This study provided compelling evidence that an early endoscopic examination is essential for a timely step-up of therapy in the case of recurrence to enhance long-term results [13].

Based on this body of evidence, different guidelines recommend a follow-up ileocolonoscopy within 6 to 12 months after surgery to evaluate for the presence of POR [37,38]. This time frame allows for an optimal assessment of disease status and aids in determining the appropriate management strategies.

The endoscopic follow-up of patients without recurrence after the first endoscopic assessment is less clear. A retrospective study of 86 patients undergoing an ICR without endoscopic evidence for recurrence at the baseline evaluation (median time of seven months) found after a median follow-up time of 3.5 years that 40.7% of patients had a late relapse (defined as either clinical recurrence, inflammatory bowel disease (IBD)-related hospitalization, occurrence of bowel damage, or need for endoscopic balloon dilatation of the anastomosis and need to repeat the surgery), with a median time to disease recurrence after baseline endoscopy of 14.2 months (interquartile range [IQR], 6.3–26.1 months) [39]. In line with these findings, the consensus guidelines from the Global Interventional Inflammatory Bowel Disease Group for the endoscopic evaluation of surgically altered bowel in IBD recommends a subsequent ileocolonoscopy for disease monitoring and that dysplasia surveillance should be performed every 1–3 years at the discretion of the treating physician [40]. On the other hand, the Spanish working group (GETECCU) recommends repeating an ileocolonoscopy in the following cases: onset symptoms with elevated biological markers, such as fecal calprotectin (FC) (>100 μg/g) or C-reactive protein (CRP), suspicion of POR on intestinal ultrasound (IUS), or progressive elevation of FC in two or three consecutive measurements, repeated every trimester [41].

**Table 1:** Rutgeerts’ score and modified Rutgeerts’ score to assess postoperative recurrence in patients with Crohn’s disease.

	Score	Endoscopic Findings
Endoscopic Postoperative Remission	i0	no lesions
	i1	≤5 aphthous ulcers
Endoscopic Postoperative Recurrence	i2	>5 aphthous ulcers with normal mucosa in-between or large lesions limited to anastomosis
	i2a (mRS)	lesions limited to the ileocolonic anastomosis (with/without anastomotic stenosis)
	i2b (mRS)	>5 aphthous ulcers or larger lesions with normal mucosa in-between the neoterminal ileum (with/without anastomotic stenosis)
	i3	aphthous ileitis with diffusely inflamed mucosa
	i4	diffuse inflammation with large ulcers, nodules and/or strictures

mRs = modified Rutgeerts’ score.

### 3.2. Cross-Sectional Image

Given the transmural nature of CD, cross-sectional imaging techniques provide a comprehensive assessment of the entire intestinal wall, thereby overcoming the inherent limitation of an endoscopy, which solely evaluates the mucosal layer [42]. IUS and magnetic resonance enterography (MRE) are non-invasive and do not expose patients to ionizing radiation, which makes them a desirable monitoring tool for postoperative CD.

A meta-analysis by Yung et al. compared the diagnostic accuracy of capsule endoscopy, MRE, and IUS for detecting endoscopic recurrence using RS in postoperative CD [43]. Five studies with 76 patients examined the diagnostic accuracy of capsule endoscopy with a sensitivity of 100%, specificity of 69%, and area under the curve (AUC) of 0.94. Capsule retention occurred in three patients (2.1%) across these studies. MRE showed a sensitivity of 97.3%, specificity of 83.7%, and AUC of 0.98, and for non-enhanced IUS, a sensitivity of 83.5%, specificity of 91.5%, and AUC of 0.93 were registered [43]. According to these findings, cross-sectional images have acceptable accuracy for monitoring POR. 

A new index for MRE in CD patients who underwent ileocecal resection, the MONITOR index, was validated in a French cohort. It is comprised of seven items, each assigned a score: 0 for the absence of MRE signs or one for the presence of wall thickening, contrast enhancement, T2 signal increase, diffusion-weighted signal increase, edema, and a diseased segment length ≥20 mm. Additionally, the presence of ulcers is assigned 2.5 points. Consequently, the score ranges from 0 (lowest probability of POR) to 8.5 (highest probability). The optimal cut-off was 1, giving a sensitivity of 79%, specificity of 55%, positive predictive value (PPV) of 68%, and negative predictive value (NPV) of 68% in predicting POR [44]. Despite good intra- and inter-reader reliability with an intraclass correlation coefficient of 0.67 and 0.67, respectively, it is not widely used. Moreover, there are inherent limitations to MRE use; it is expensive, has limited availability, is time-consuming, and requires oral and intravenous contrast. 

IUS has advantages over other techniques, such as accessibility, immediacy, non-invasive nature, absence of radiation, and low cost, and it is very well tolerated by patients [45]. Additionally, diagnostic accuracy might improve with different ultrasonographic contrast methods, including small intestine contrast ultrasound (SICUS) and contrast-enhanced ultrasonography (CEUS), which have been compared to “simple” IUS [46]. SICUS is based on the use of an oral contrast solution (polyetilenglycol) and can evaluate minor changes in small bowel walls and established CD complications, such as strictures with potential prestenotic dilatation [47]. Otherwise, CEUS requires intravenous contrast media, allowing for the evaluation of the microvasculature and a better assessment of the intestinal wall vasculature, which is a sensitive tool for estimating the inflammatory activity of the disease [48]. However, the limited increase in sensitivity achieved using CEUS or SICUS must be weighed against the added invasiveness and time constraints involved.

A meta-analysis of ten prospective studies by Rispo et al. evaluated the accuracy of IUS in detecting POR. A bowel wall thickness (BWT) >3 mm showed a sensitivity of 82%, specificity of 88%, and overall accuracy of 87.5% for diagnosing endoscopic recurrence. SICUS sensitivity was 99%, specificity was 75%, and overall accuracy was 92%. A cut-off value of BWT ≥ 5.5 mm at IUS revealed a sensitivity of 83.8%, specificity of 97.7%, negative likelihood ratio of 0.165, and positive likelihood ratio of 36.4 for severe endoscopic recurrence (RS ≥ 3) [47].

A more recent multicenter prospective study assessed a non-invasive approach combining IUS and FC. Ninety-one consecutive patients underwent an ileocolonoscopy and IUS within one year of surgery and with no more than three months of difference between both examinations. FC, CRP, and clinical activity were collected at the time of IUS. BWT per 1-mm increase (odds ratio [OR], 2.43; 95% CI, 1.21–4.89; *p* = 0.012), the presence of mesenteric lymph nodes (OR, 15.63; 95% CI, 1.48–164.54; *p* = 0.022), and FC values ≥ 50 μg/g (OR, 8.58; 95% CI, 2.45–29.99; *p* < 0.001) were all identified by multivariable analysis as independent predictors for endoscopic recurrence. A BWT ≥ 3 mm and FC ≥ 50 μg/g or the presence of lymph nodes correctly classified 75% and 56% of patients, respectively, with less than 5% of patients falsely classified as having endoscopic recurrence [49]. These findings support that IUS and FC are reliable, non-invasive tools and could have a great impact on the follow-up of CD patients after ileocecal resection, avoiding colonoscopy in some cases. However, larger prospective studies are needed.

### 3.3. Biomarkers

Even though colonoscopy is the gold standard, it is invasive, requires bowel preparation, is time-consuming, expensive, cannot be easily repeated, and is not devoid of complications [50]. In contrast, stool and serum sampling are well-tolerated and excellent tools to stratify patients according to their risk of recurrence and tailor recommendations for colonoscopies [49,51].

#### 3.3.1. Fecal Calprotectin

Calprotectin is a calcium- and zinc-binding protein that constitutes 60% of the neutrophil cytosolic protein [52]. FC is a non-invasive marker of gut inflammation with a strong correlation with endoscopic and histological inflammation, which is helpful in the diagnosis and follow-up of patients with IBD [53,54]. Its limitations encompass challenges in the pre-analytical stage, such as the timing of sample collection, stool storage, and variations in stool weight, as well as analytical difficulties related to the types of measurement kits used. These factors can influence the results, leading to heterogeneous findings both intra-individually and across different centers [55].

As FC is a reliable predictor of active inflammation, it allows for the differentiation of active and inactive disease, prediction of possible relapses, and monitors response to treatment [56,57,58,59,60,61,62,63]. A meta-analysis published in 2018 assessed the yield of FC for predicting endoscopic recurrence. Most of the studies were prospective and defined recurrence as RS > i2. The best diagnostic accuracy was obtained for an FC value of 150 μg/g, with a pooled sensitivity of 70% (95% CI 59–81%) and specificity of 69% (95% CI 61–77%) [64], but it is important to consider that there were different thresholds for the time of endoscopy evaluation, with only three of the nine studies performing it in the first year after surgery [65,66,67] and none of the studies during performed it the first six months, as recommended in recent guidelines. 

A study by Boschetti et al. with 86 patients and examinations performed after a mean interval of 8.2 ± 0.5 months found that a cut-off point of 100 μg/g was the most effective in distinguishing between endoscopic remission and recurrence, with a sensitivity of 95%, specificity of 54%, positive and NPVs, as well as overall accuracies of 69%, 93%, and 77%, respectively [67]. By using this cut-off, 4.7% of patients who were in endoscopic recurrence had an FC ≤ 100 μg/g and subsequently would not have been correctly stratified on the basis of only an FC measurement. In addition, the sole assessment of FC concentration in the postoperative setting would have been able to accurately differentiate patients as having no signs of endoscopic recurrence in the vast majority, potentially allowing the avoidance of 30% of endoscopic examinations in the mentioned cohort. Nevertheless, it is crucial to acknowledge that alternative studies do not endorse FC as an adequate method for monitoring patients with POR [66]. Consequently, the focus should be on assessing the trend of FC values over time rather than relying on a single value alone when utilizing FC to monitor the progression of these patients. 

There is the valid question of when FC ceases to show the inflammatory state typical of surgery and becomes an inflammatory marker of reactivation or recurrence of the disease. A small cohort of 13 patients was followed prospectively for one year with regular FC and lactoferrin measurements. Patients without complications after ICR normalized stool markers two months after surgery [68]. The POPCUR is a randomized, placebo-controlled, postoperative prevention trial assessing curcumin vs. placebo after 21 days of an azathioprine course post-surgery. Fecal samples (FC and coproculture) were taken at baseline, one month, and three months postoperatively. In addition to the level of FC, the relative variation between two of the time points (expressed as a percentage) was assessed as a predictor of endoscopic POR (modified RS ≥ i2b). There were no differences in median FC levels between patients with and without postoperative recurrence (baseline *p* = 0.15; one month *p* = 0.44; three months *p* = 0.28). The researchers calculated the relative variation between the level of FC at baseline and three months and determined that an increase (Δ FC M3-M0) of more than 10% demonstrated the best performance to predict endoscopic POR at six months, with a sensitivity of 64.7%, specificity of 87.5%, NPV of 77.8%, and PPV of 78.6% [51]. 

Given these aforementioned results, the ECCO workshop recommendation is to begin measuring FC levels three months after surgery and eventually anticipate endoscopic assessment according to its levels and trend in the follow-up [15].

#### 3.3.2. Serum Biomarkers

Evidence indicates adherence is often higher for blood-based tests vs. stool-based assays like FC, conferring advantages in terms of patient acceptance and real-world completion rates. [69]. Additionally, the low disease burden in early recurrence and the inconsistent elevation of CRP levels make CRP measurement insensitive to detecting early disease recurrence [70]. Novel biomarkers have emerged in recent years, including the development and validation of the endoscopic healing index (EHI), a blood test that measures 13 serum proteins that allow for the adequate identification of patients with endoscopic remission (SES-CD ≤ 2) [71]. The EHI was validated in 278 CD patients divided into two cohorts (116 biologic-naive with early-stage disease and 195 biologic-exposed with chronic disease) by comparing its scores, ranging from 0–100, with higher values correlating to more severe endoscopic activity. A cut-off value of 20 points identified patients in remission with the highest level of sensitivity (83.2% and 97.1% for each validation cohort, respectively), with specificity values of 36.6% and 69.0%, respectively [71]. A subsequent study using the EHI in serum samples of patients from the aforementioned POCER trial [13] evaluated the overall accuracy of the tool at various cut-offs for the presence of endoscopic recurrence (RS ≥ i2). At six months, the EHI < 20 and FC < 100 μg/g had similar sensitivity (81.8% and 90.9%, respectively) and NPV (84.0% and 91.7%, respectively) for the detection of endoscopic recurrence. However, at 18 months, the EHI could not accurately discriminate between remission and recurrence, unlike FC, with an NPV of 64.9% vs. 89.7%, respectively [72]. The authors pointed out that these results could be related to specific markers within the EHI, such as those of matrix remodeling, being more relevant early in the postoperative period. 

Nowadays, there is no recommendation to replace FC with these serum markers. Further research is still warranted to characterize their predictive performance and cost-effectiveness, as existing evidence has primarily assessed their correlation with established tests rather than validating their independent accuracy in guiding clinical management.

## 4. Risk Factors for Postoperative Recurrence

**Figure 1:**
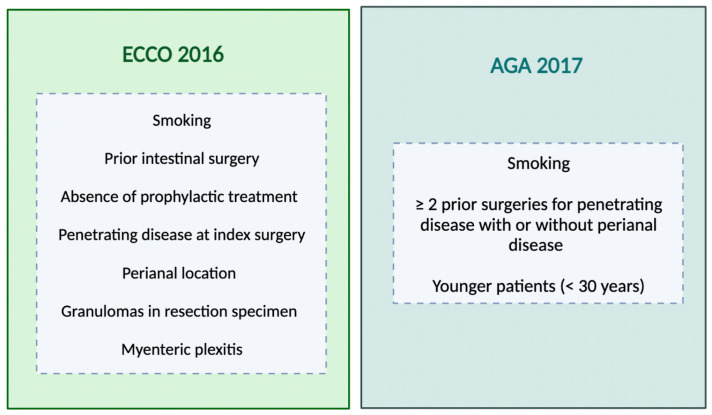
Risk factors for postoperative recurrence according to different societies’ guidelines. ECCO = European Crohn’s and Colitis Organization; AGA = American Gastroenterological Association.

Postoperative monitoring is important for detecting recurrence; however, not all patients experience changes warranting medical intervention [73]. Close follow-up permits finding patients with progressive disease activity that may benefit from optimized management [74]. The evaluation of a patient’s risk factors is important when determining the need for prophylactic therapy after CD surgery [75,76] (Figure 1). 

Cigarette smoking has been consistently shown to robustly predict both surgical (OR = 2.56; 95% CI 1.79–3.67; *p* < 0.001) and clinical (OR= 2.15; 95% CI 1.42–3.27; *p* < 0.001) recurrence compared to non-smokers [77]. A younger age (<30 years) and longer disease duration have been hypothesized as potential predictive factors, but evidence regarding their impact is mixed [78]. Consequently, some guidelines consider these as high-risk attributes warranting prophylaxis, while others focus primarily on smoking status given its clear evidence base [37,77]. 

A cohort of 34 patients who underwent ileocolonic resection demonstrated that those with penetrating disease had a significantly increased risk of early clinical recurrence (<3 years after index operation) in contrast with none of the stricturing phenotype patients [79]. Additionally, a meta-analysis encompassing 13 studies and a total of 3044 patients determined that a perforating phenotype was significantly associated with a higher risk of POR (hazard ratio [HR] = 1.50, 95% CI 1.16–1.93, *p* = 0.002) [80]. Due to this evidence, a penetrating phenotype is an important risk factor considered to be associated with early POR. 

History of past resection [81,82] and perianal disease are also recognized as predictors of POR [37,78,83]. Histological risk factors have been identified, and the most studied predictor to date is the presence of granulomas in the surgical specimen. In a meta-analysis by Similis et al., including 22 studies, granulomatous CD increased the risk of recurrence (OR, 1.37, 95% CI 1.02–1.84, *p* = 0.04) and reoperation (OR, 2.38, 95% CI 1.43–3.95, *p* < 0.001) [84]. More recently, a study including 418 patients who underwent initial ICR with >4 years of follow-up did not find a difference in the endoscopic recurrence between the presence or absence of granulomas, but the first was independently associated with a higher risk of subsequent surgery [85]. Myenteric plexitis (defined as the presence of at least one inflammatory cell in an enteric ganglion or nerve bundle) at the proximal resection margin was a predictive factor for early endoscopic recurrence at 3 and 12 months; however, more research is required to fully understand these findings [86]. Regarding the patient’s risk profile, a multicenter Italian study compared the outcomes of 195 CD patients with one risk factor for POR who either received immediate immunosuppression (prophylaxis group) or treatment guided by endoscopy findings (endoscopy-driven group) following ICR [87]. No significant differences were observed between the approaches regarding endoscopic (36.1% vs. 45.5%, *p* = 0.10) or clinical recurrence (17.9% vs. 34.8%, *p* = 0.09) rates at 12 months. These findings suggest that endoscopy-guided management may be as effective as prophylactic immunosuppression for patients with a single risk factor.

## 5. Prophylaxis of Postoperative Recurrence

The main guidelines vary regarding the initiation of prophylaxis. The ECCO guidelines recommend starting it in patients with risk factors for recurrence [83], while the American Gastroenterological Association (AGA) guidelines recommend starting early generalized prophylaxis; however, with a comment on patients who are at low risk of POR who may prefer to avoid the small risks of adverse effects from prophylactic medications over the potential risk of recurrence. In this case, they may opt for endoscopy-guided pharmacological treatment rather than prophylaxis [37]. 

Randomized controlled trials (RCTs) evaluated the potential of various drugs in preventing POR (Table 2), with the primary endpoint being endoscopic recurrence as assessed by the RS [88]. Different studies have shown that endoscopic POR can occur as early as weeks after surgery [18]. Therefore, prophylactic therapy should begin soon after surgery to limit the development of endoscopic postoperative lesions. However, the optimal strategy, whether “step-up” or “top-down”, for preventing endoscopic and clinical recurrence remains undetermined, as both approaches have their respective advantages and potential risks.

### 5.1. Antibiotics

Antibiotics have been shown to be effective in reducing POR by limiting anaerobic bacterial overgrowth and inflammation in the intestinal mucosa [89]. 

A placebo-controlled randomized trial was conducted to assess the efficacy of metronidazole given at 20 mg/kg/day vs. placebo for the first three months after ICR. A lower rate of endoscopic (52% vs. 75%, *p* = 0.09) and histological (17% vs. 54%, *p* = 0.008) POR in the metronidazole group was evident. Nevertheless, adverse events (including gastrointestinal intolerance, limb paraesthesia, abnormal liver function, leukopenia, polyneuropathy, and psychosis) were more frequent, leading to treatment discontinuation in about 30% of patients [90]. As a result, given the high rate of intolerance and the possibility of developing severe adverse reactions, its use in clinical practice is greatly reduced.

Ornidazole, an antibiotic that belongs to the same family as metronidazole, has also been studied in this context; however, the rate of withdrawal due to adverse events was higher than placebo (12/38 vs. 5/40; *p* = 0.041) [91]. Conversely, ciprofloxacin showed no significant difference in the rate of endoscopic POR compared to placebo (65% vs. 69%, *p* < 0.805) [92].

### 5.2. Mesalazine

The role of mesalazine has been investigated, considering the potential adverse events associated with other drug classes and the long-term practicality of using this medication [93]. Several studies have used different doses of mesalazine to prevent clinical POR. A meta-analysis of five studies, including 730 patients, showed that mesalazine is more effective than a placebo in preventing clinical POR. During a follow-up period ranging from 12 to 72 months, the relapse rate was 36% in patients treated with mesalazine compared to 43% of the placebo group (RR, 0.83, 95% CI 0.72–0.96). The number needed to treat (NNT) to prevent clinical relapse was 13 [94]. There were only three studies included in the analysis that reported endoscopic recurrence, and the results were not consistent. Due to substantial heterogeneity and a high risk of bias, the evidence on the effect of 5-ASA on endoscopic recurrence is uncertain. Interestingly, higher doses did not increase its efficacy [94]. 

### 5.3. Corticosteroids

Budesonide as prophylaxis was evaluated in two RCTs [95,96]. The study by Hellers et al., a double-blind RCT, evaluated the efficacy of oral budesonide in preventing endoscopic recurrence at a fixed dose of 6 mg per day vs. placebo. An ileocolonoscopy was performed after 3 and 12 months, and the frequency of endoscopic recurrence did not differ between the groups at either time point [63]. Ewe et al. conducted a multicenter trial and did not find a significant difference in endoscopic nor in clinical recurrence rate after one year between patients taking budesonide and those taking placebo [95]. 

### 5.4. Immunomodulators 

The efficacy of thiopurines prophylaxis for POR has been assessed in several RCTs [97,98,99]. Hanauer et al. conducted a multicenter, double-blind, double-dummy trial with 131 patients who were randomized to receive 6-mercaptopurine (50 mg), mesalazine (3 g), or a placebo. Clinical recurrence at 24 months was 50% (95% CI, 34–68%), 58% (95% CI, 41–75%), and 77% (95% CI, 61–91%) in patients receiving 6-mercaptopurine, mesalazine, and placebo, respectively. Endoscopic recurrence rates were 43% (95% CI, 28–63%), 63% (95% CI, 47–79%), and 64% (95% CI, 46–81%), respectively. The 6-mercaptopurine was more effective than a placebo (*p* < 0.05) in preventing clinical and endoscopic recurrence over two years [98]. In the study by D’Haens et al., all patients received metronidazole for three months together with azathioprine (AZA) or placebo for 12 months. The endoscopic recurrence at one year was significantly lower with AZA (43.7% vs. 69%; *p* = 0.048) [99]. The TOPPIC trial was a double-blinded, placebo-controlled RCT, with 128 patients randomized to 6-mercaptopurine at a dosage of 1 mg/kg daily and 120 patients to placebo. A total of 13% of patients treated with 6-mercaptopurine vs. 23% of patients treated with placebo had a clinical POR after three years, but the difference was not statistically significant [100]. There have been different published meta-analyses [101,102] with similar results. The most recent concluded that thiopurines were more effective than a placebo in preventing clinical POR after 12–36 months of follow-up (51% vs. 64%, RR 0.79; 95% CI 0.67–0.92) without a difference in endoscopic recurrence, defined as RS ≥ 2 (RR 0.85; 95% CI 0.64–1.13) [102].

Meta-analysis comparing thiopurines vs. mesalazine did not find any difference in the prevention of clinical relapse at 24 months, approximately 65% vs. 60% for thiopurines and mesalazine, respectively [94,102]. Nonetheless, the efficacy of AZA was slightly superior to that of mesalazine in preventing endoscopic relapse (RR, 0.78; 95% CI 0.52–1.17) [102].

### 5.5. Anti-Tumor Necrosis Factor

Few studies have assessed the role of anti-tumor necrosis factor (anti-TNF) in the prevention of POR. In the PREVENT trial, the efficacy of infliximab in preventing POR was evaluated in 297 patients who had undergone ICR. Patients with at least one risk factor were randomly assigned to receive infliximab (IFX) or placebo every 8 weeks for 200 weeks. The primary endpoint was clinical recurrence and endoscopic recurrence or development of a new or re-draining fistula or abscess before or at week 76. The results showed that infliximab significantly reduced endoscopic recurrence compared to placebo (30.6% vs. 60.0%; *p* < 0.001). However, it did not achieve the first outcome; the reduction in clinical recurrence was not statistically significant (12.9% vs. 20.0%; *p* = 0.097). Patients previously treated with anti-TNF agents or those with more than one resection were at greater risk for clinical recurrence. It is important to highlight that patients assigned to the infliximab group did not receive the classic induction schedule (i.e., 5mg/kg every 8 weeks dosing without the 3-dose induction regimen) [103]. 

Savarino et al. conducted an RCT with 51 patients and compared adalimumab 160/80 mg at 0 and 2 weeks, followed by 40mg every two weeks, with AZA at a dose of 2 mg/kg every day or mesalazine at a dose of 3 g/day for 24 months. Endoscopic POR was 6.3% in patients treated with adalimumab, compared to 64.7% in the AZA group (OR 0.036; 95% CI 0.004–0.347) and 83.3% in the mesalazine group (OR 0.013; 95% CI 0.001–0.143). The clinical recurrence was significantly lower in the adalimumab group compared to the AZA and mesalazine groups [104].

In recent years, several meta-analyses have been published evaluating the efficacy of anti-TNF agents in preventing postoperative clinical and endoscopic recurrence, mostly showing a greater efficacy on both outcomes compared to therapies such as mesalazine and AZA [105,106,107]. Beelen et al. conducted a meta-analysis of individual participant data from six RCTs, reporting that anti-TNF were superior to thiopurines in preventing endoscopic (RR 0.52, 95% CI 0.33–0.80) and clinical POR (RR 0.50; 95% CI, 0.26–0.96) as well as severe endoscopic recurrence (RR 0.41; 95% CI, 0.21–0.79), confirming the superiority of anti-TNF in preventing both endoscopic and clinical POR after ICR. Previous exposure to anti-TNF and penetrating disease behavior was associated with an increased risk of endoscopic recurrence [108]. Furthermore, an RCT from Reguiero et al. evaluated patients for at least five years post-intestinal resection [109]. Patients who initially received IFX showed significantly longer durations until endoscopic recurrence (123 ± 747 days vs. 460 ± 121 days, *p* = 0.003) and surgical recurrence (1798 ± 359 days vs. 1058 ± 529 days, *p* = 0.04) compared to those who received placebo. Colonoscopy findings and rates of achieving endoscopic remission markedly favored long-term infliximab therapy (22.2% vs. 93.9%, *p* < 0.0001). Additionally, time to reoperation was meaningfully extended in the IFX cohort (*p* = 0.047). A systematic review and meta-analysis compared the efficacy of ADA and IFX in preventing POR [110]. Three studies including 268 patients were analyzed, and no significant differences were found in the total endoscopic recurrence rates (27.1% vs. 32.3%, OR 0.696, 95% CI 0.403–1.201, *p* = 0.193) or at one year (OR 0.799, 95% CI 0.329–1.940, *p* = 0.620). Clinical recurrence rates also did not differ significantly between drugs (OR 0.477, 95%CI 0.477–1.712, *p* = 0.755) [110]. The effect of preoperative anti-TNF therapy on postoperative infection risk was examined in the PUCCINI trial, in which 947 IBD patients underwent surgery, and anti-TNF use within the previous 12 weeks was reported in 382 individuals [111]. Any infection rates were similar in exposed (18.1%) vs. unexposed patients (20.2%, *p* = 0.469), as were surgical site infections (SSIs) rates (12.0% vs. 12.6%, *p* = 0.889). Multivariable analysis found that anti-TNF therapy was not associated with a higher risk of infection (OR 1.050, 95%CI 0.716–1.535) or SSI (OR 1.249, 95%CI 0.793–1.960) [111]. Moreover, continuing anti-TNF therapy to prevent POR may help limit anti-drug antibody development. [12] In a French prospective study evaluating preoperative anti-TNF levels, IFX and ADA concentrations >1 mg/mL (OR 0.69, 95% CI 0.21–2.22) or >3 mg/mL (OR 0.95, 95% CI 0.28–2.96) after ileocolic resection were not significantly associated with a higher postoperative complication risk [112].

Another point of discussion in the management of POR in CD concerns the question of whether to use a ‘top-down’ or ‘step-up’ strategy. A retrospective study compared POR in 115 CD patients receiving top-down vs. step-up biologics after ICR [73]. The propensity score analysis showed that a top-down strategy (ADA/IFX/Ustekinumab [UST]) initiated within one month of surgery was more effective at preventing endoscopic recurrence (RS ≥ i2a) at a 6-month colonoscopy (46.8% vs. 65.9%, *p* = 0.042) and achieving complete remission (RS i0; 45.3% vs. 19.3%, *p* = 0.004). Endoscopic recurrence correlated with higher risks of clinical recurrence (HR 1.97, *p* = 0.029) and bowel damage progression (HR 3.33, *p* = 0.018). Among patients without endoscopic recurrence, top-down reduced risk of these outcomes (clinical recurrence HR 0.59, *p* = 0.025; bowel damage progression HR 0.73, *p* < 0.001) [73]. Another study compared top-down vs. step-up ADA strategies in 120 pediatric patients over 24 months [113]. At the endline, remission was higher in the top-down group (73% vs. 51%, *p* < 0.01). A propensity analysis found top-down to be more effective in maintaining remission (HR = 0.36, 95% CI 0.15–0.87, *p* = 0.02). Top-down mainly used monotherapy vs. combination in step-up (*p* < 0.001) [113]. Previous results may be indirectly confirmed by a retrospective Scottish study, which found 5-year surgery risks fell from 20.4% to 13.0% (*p* < 0.001), while biologic use rose from 5.7% to 44.9% (*p* < 0.001) from 2000–2004 to 2014–2017 [114]. Increased earlier biologic initiation corresponded to lower surgery rates, suggesting top-down or accelerated step-up approaches may reduce post-surgical need in newly diagnosed CD [114].

### 5.6. Vedolizumab and Ustekinumab 

Data on the use of vedolizumab (VDZ) and UST are limited, and the evidence is only just beginning to emerge. 

Recently, the preliminary results of the REPREVIO trial, a prospective placebo-controlled RCT investigating the efficacy of VDZ in preventing POR in patients with at least one risk factor (active smoking, prior resection, prior surgery for perforating complication, or previous exposure to anti-TNF) have been reported. Endoscopic remission (RS i0) was observed in 18/43 patients on VDZ vs. 1/37 on placebo (42% vs. 3%, *p* < 0.001, respectively) [115]. A retrospective multicenter study was conducted to evaluate the effectiveness of early prophylaxis (within six months since surgery) with biological therapy and compared anti-TNF therapy to VDZ and UST in a real-world setting [116]. The study included 297 patients with no significant difference by groups in endoscopic POR rates within one year: anti-TNF 40.2%, VDZ 33%, and UST 61.8%. Patients treated with VDZ and UST were more biologic-experienced with higher rates of previous surgery. After controlling for confounders, no differences in the endoscopic POR risk were seen between anti-TNF prophylaxis and other groups, and combining immunomodulators was not associated with a lower endoscopic POR [116]. The results of a retrospective ENEIDA cohort study involving 40 patients treated with UST and 25 with VDZ for the prevention of POR were similar to these data. The study showed that within 18 months of surgery, the incidence of endoscopic POR was 40% for VDZ and 42% for UST [117]. Also, another retrospective study compared UST to AZA for preventing POR in 63 patients [118]. After a propensity score adjustment, endoscopic recurrence at six months was lower with UST (28%) vs. AZA (28 vs. 54.5%, *p* = 0.029). Severe recurrence rates were also lower with UST (16.9% vs. 27.9%). Another retrospective study examining 203 post-surgical CD patients undergoing surgery registered that 22 received VDZ, while 58 received anti-TNF therapy [119]. Endoscopic remission at 6–12 months was lower with VDZ (25%) vs. anti-TNF (66%, *p* = 0.01). VDZ was associated with a 5-fold increased recurrence risk (OR 5.77, 95% CI 1.71–19.4, *p* = 0.005). Propensity score-matched analysis also showed lower remission with VDZ (25% vs. 69%, *p* = 0.03).

On the other hand, a retrospective cohort study of 141 CD patients who underwent surgery found that 45.2% of those on postoperative biologics (ADA 22.0%; IFX 9.2%, UST 2.8%, VDZ 2.1%) had endoscopic recurrence at 6–12 months vs. 80.8% who were not on biologics (*p* < 0.0001) [120]. Over a median 28-month follow-up, 0% on biologics were hospitalized or required repeat surgery vs. 12.1% who were not on biologics (*p* = 0.01). The no biologics group had a >23.3% 5-year rate of hospitalization/surgery (*p* = 0.0221) and >49.7% 5-year medical escalation rate (*p* = 0.0013) compared to the biologics group. The absence of postoperative biologics was an independent risk factor for endoscopic recurrence (OR 0.22, 95% CI 0.1–0.51; *p* = 0.0004).

**Table 2:** Selected evidence about pharmacological prophylaxis of postoperative recurrence in patients with Crohn’s disease.

Authors	Drug	Study Design	Population	AGE	Sex (M/F)	Risk Factors	Outcomes	Results
Herfarth et al. (2013) [92]	Ciprofloxacin vs. placebo	RCT	33 patients	30 (18–70)	18/15	Smo = 12% nR = 18% Str = 54%	Endoscopic POR 6 months (65% vs. 69%, *p* < 0.805)	Ineffective
Rutgeerts et al. (2005) [91]	Ornidazole vs. placebo	RCT	78 patients	33 (18–70)	42/16	Smo = 17% nR = 24% Str = 18%	Clinical POR 1 year (8% vs. 38%, *p* = 0.046); Endoscopic POR 1 year (79% vs. 54%, *p* = 0.037)	Effective
Rutgeerts et al. (1995) [90]	Metronidazole vs. placebo	RCT	60 patients	25	NA	nR = 41% Str = 58%	Endoscopic POR 3 months (52% vs. 75%, *p* = 0.09); Histological POR 3 months (17% vs. 54%, *p* = 0.008)	Effective
Hellers et al. (1999) [95]	Budesonide 6 mg/d vs. placebo	RCT	129 patients	35 (17–81)	62/67	nR = 28%	Endoscopic POR 3 months (52% vs. 31%, *p* = NA); Endoscopic POR 12 months (58% vs. 52%, *p* = NA)	Ineffective
Ewe et al. (1999) [96]	Budesonide 3 mg/3 d vs. placebo	RCT	62 patients	34 (23–47)	37/46	nR = 62%	Clinical and/or endoscopic POR 1 year 1 (57% vs. 70%)	Ineffective
NA	MTX	NA	NA	NA	NA	NA	NA	No data
Gjuladin-Hellon et al. (2019) [102]	Thiopurines vs. placebo	Meta-analysis 3 studies	408 patients	NA	NA	NA	Reduction endoscopic POR 12–36 months (67% vs. 75%, RR 0.85, 95% CI 0.64–1.13); Reduction clinical POR 12–36 months (51% vs. 64%, RR 0.79, 95% CI 0.67–0.92)	Effective
Peyrin-Biroulet et al. (2009) [101]	Thiopurines vs. placebo/ metronidazole/ 5-ASA	Meta-analysis 4 studies	433 patients	NA	NA	NA	Reduction endoscopic POR (mean difference 15%, 95% CI 1.8–29%, *p* = 0.026); Reduction clinical POR (mean difference 8%, 95% CI: 1–15%, *p* = 0.021)	Effective
Reguiero et al. (2016) [103]	IFX vs. placebo	RCT	297 patients	36 (18–73)	158/139	nR = 42% Str = 57%	Clinical POR 76 weeks (12.9% vs. 20%, *p* = 0.097); endoscopic POR 76 weeks (30.6% vs. 60%, *p* < 0.002)	Effective
De Cruz et al. (2015) [13]	Metronidazole+ Thiopurines/ ADA vs. Metronidazole	RCT	118 patients	36 (26–47)	19/33	Smo = 31% nR = 31% Str = 40%	Endoscopic POR 6 months (52% vs. 75%, *p* = 0.03)	Effective
Buisson et al. (2021) [118]	UST vs. AZA	Observational Retrospective	63 patients UST (32); AZA (31)	37	15/48	Smo = 38%	Endoscopic POR 6 months (28% vs. 54.5%, *p* = 0.029)	Uncertain
Yanai et al. (2022) [116]	UST/VDZ vs. Anti-TNF	Observational Retrospective	297 patients UST (34)/VDZ (39) vs. anti-TNF (224)	24 (IQR 19–32)	166/131	Smo = 36% nR = 2% Str = 64%	Endoscopic POR 1 year 41.8%; UST and anti-TNF (OR 1.86, 95% CI 0.79–4.38)	Uncertain
Mañosa et al. (2022) [117]	UST	Observational Retrospective	40 patients	34 (24–55)	25/15	Smo = 22% nR = 42% Str = 37%	Clinical POR 12 months in 32%; Endoscopic POR 18 months in 42%	Uncertain
Yamada et al. (2018) [119]	VDZ vs. anti-TNF	Observational Retrospective	80 patients VDZ (22); anti-TNF (58)	33	38/42	Smo = 12% Str = 42%	Endoscopic remission 6–12 months (25% vs. 66%, *p* = 0.01)	Uncertain
Mañosa et al. (2022) [117]	VDZ	Observational Retrospective	25 patients	38 (31–62)	15/10	Smo = 16% nR = 40% Str = 52%	Clinical POR 12 months in 30%; endoscopic POR 18 months in 40%	Uncertain

RCT = randomized controlled trial; MTX = methotrexate; M = male; F = female; Smo = smokers; nR = number of resections > 1; Str = stricutring phenotype; POR = postoperative recurrence; AZA = azathioprine; TNF = tumor necrosis factor; IFX = infliximab; ADA = adalimumab; UST = ustekinumab; VDZ = vedolizumab; IQR = interquartile range; RR = relative risk.

## 6. Medical Treatment of Postoperative Recurrence

Nowadays, the evidence base for guiding the medical treatment of POR in CD patients is still limited (Table 3). Further research is greatly needed to define the most effective strategies for prophylaxis and management of these clinical scenarios.

### 6.1. Antibiotics

Currently, there is no evidence justifying the use of antibiotics in the treatment of POR [121].

### 6.2. Mesalazine

Two RCTs have yielded insights into the potential limited advantage of mesalazine in the management of POR [122,123]. In an RCT conducted by Reinisch et al., 78 patients showing endoscopic evidence of CD recurrence, without clinical symptoms of POR, were enrolled to compare the effectiveness of mesalazine to AZA. Overall, the incidence of treatment failure, defined as either clinical recurrence or drug discontinuation within one year, was relatively low and comparable between the mesalazine and AZA groups (11% vs. 22%; *p* = 0.19, respectively) [122]. Orlando et al. reported no significant differences in 46 patients with severe endoscopic recurrence in therapeutic failure rates after one year of treatment with either a high dose of mesalazine or AZA (21% vs. 14%; *p* = 0.702, respectively) [123]. Additionally, mesalazine-treated patients exhibited less frequent endoscopic improvement after one year compared to their AZA counterparts (RS reduction 8–34% vs. 36–63%, respectively). Furthermore, a small case-control study failed to demonstrate any benefit from the addition of mesalazine in patients with subclinical endoscopic recurrence who were already receiving thiopurine therapy [124]. 

### 6.3. Corticosteroids

Unlike prophylaxis, there is currently no data on the use of corticosteroids in the treatment of POR [121]. 

### 6.4. Immunomodulators

The effectiveness of AZA in treating CD recurrence has been known since the first retrospective study by D’Haens et al. [125]. The two aforementioned RCTs [122,123] highlighted the superiority of AZA over mesalazine in the prevention of clinical recurrence and the reduction of RS in asymptomatic patients with endoscopic POR. Dissimilarly, another RCT conducted on 63 patients receiving AZA in the first two weeks after surgery or in the case of POR did not show significant differences in treatment strategy; endoscopic remission was achieved by 50% in the systematic group and 42% in the endoscopy-driven group (*p* = 0.521) [126]. To date, there is a lack of evidence on the use of methotrexate and thioguanine in POR treatment.

### 6.5. Anti-Tumor Necrosis Factor 

To date, the existing literature supports the efficacy of anti-TNF therapy in POR treatment. A meta-analysis conducted by Carla-Moreau et al. demonstrated IFX to be superior to control groups in reducing endoscopic POR based on a small pooled sample (*n* = 50), albeit with a wide confidence interval (OR 16.64; 95% CI 2.51–110.27, *p* < 0.004; number needed to treat = 2.3) [105]. Unfortunately, there was no sufficient data to examine clinical POR. In the POCER trial, 33 patients who initially received AZA switched to adalimumab due to endoscopic recurrence after six months. At 18 months, 39% of the patients stepped up to adalimumab (*n* = 13/33) reached endoscopic remission [127]. A monocentric prospective study evidenced that adalimumab treatment for the endoscopic POR resulted in complete (RS i0, *n* = 3) or near-complete (RS i1, *n* = 6) endoscopic remission in addition to clinical remission in 56% of patients after 24 months [128].

An extensive retrospective study evaluating anti-TNFs involving 83 patients treated with IFX and 96 with adalimumab showed 61% improvement in endoscopic outcomes, i.e., any decrease from baseline RS [129]. Notably, 42% of patients achieved complete endoscopic remission. Compared to adalimumab, concomitant use of thiopurines and IFX treatment seem to be associated with endoscopic improvement (OR = 2.15, 95% CI 1.04–4.46; *p* = 0.03, and OR 2.34, 95% CI 1.18–4.62; *p* < 0.01, respectively) and endoscopic remission (OR 3.16, 95% CI 1.65–6.05; *p* < 0.01 and OR 2.01, 95% CI 1.05–3.88; *p* = 0.04, respectively). However, about 30% of the patients were already treated with anti-TNFs before surgery, and almost half of them continued the same drug; nevertheless, this did not affect the post-surgical outcomes [129]. Lastly, observational studies conducted in a real-world setting confirmed that anti-TNF therapy is the most effective strategy for treating endoscopic recurrence in CD [130].

### 6.6. Ustekinumab

In a case series with 15 patients with POR treated with UST, clinical remission was achieved in 12/15 cases, while 11/11 achieved endoscopic remission [131]. Recently, a retrospective study investigated UST treatment in 44 patients [132]. At a baseline colonoscopy 6–12 months after ICR, 75% exhibited severe recurrence (RS i3/i4). Following 14.5 ± 5.5 months of treatment, endoscopic response (≥1-point RS reduction) occurred in 50% of patients; 27.3% achieved remission (RS i0/i1). At 17.8 ± 8.4 month follow-up, clinical response was seen in 72.7% of patients, and all non-responders (27.2%) failed to achieve an endoscopic response [132]. However, a retrospective study—lacking a clear POR definition—compared UST to anti-TNF therapy (48 vs. 57, respectively). UST showed lower efficacy in obtaining clinical remission (40% vs. 61%, *p* = 0.08), objective remission (42% vs. 71%, *p* = 0.01)—defined as endoscopic remission—SES-CD < 3, modified RS ≤ i2a, or absence of ulcers—or biochemical remission—FC < 150 μg/g or PCR < 1 mg/dL. Deep remission, referred to as clinical and objective remission, was also lower in the UST group (16% vs. 44% *p* = 0.008) [133]. 

### 6.7. Vedolizumab

A retrospective study conducted on 58 patients treated with VDZ due to endoscopic recurrence showed endoscopic success, considered as a decrease of RS by >1 point, in 47.6% of cases [134]. Clinical failure was observed in 19% after one year and 32.8% at the end of follow-up (mean 24.8 ± 13.1 months), while seven patients (12.1%) required new surgery.

**Table 3:** Selected evidence about the pharmacological treatment of postoperative recurrence in patients with Crohn’s disease.

Authors	Drug	Study Design	Population	AGE	Sex (M/F)	Risk Factors	Outcomes	Results
NA	ANTIBIOTICS	NA	NA	NA	NA	NA	NA	No data
NA	STEROIDS	NA	NA	NA	NA	NA	NA	No data
NA	MTX	NA	NA	NA	NA	NA	NA	No data
Reinisch et al. (2010) [122]	5-ASA vs. AZA	RCT	78 endoscopic POR	35 (17–81)	62/67	nR = 28%	Treatment failure at 1 year (11% vs. 22%, *p* = 0.19); endoscopic improvement (34.4% vs. 63.3%, *p* = 0.023); clinical POR more often with mesalazine	Uncertain
Orlando et al. (2020) [123]	5-ASA vs. AZA	RCT	48 endoscopic POR	34 (23–47)	30/36	Smo = 43% Str = 87%	Treatment failure at 1 year (21% vs. 14%, *p* = 0.7); endoscopic improvement (8.3% vs. 36.4%, *p* = 0.035); clinical POR more often with mesalazine	Uncertain
Carla-Moreau et al. (2015) [105]	IFX vs. AZA/5-ASA	Meta-analysis	50 endoscopic POR	36	NA	NA	IFX is more effective for endoscopic POR (OR 16.6; 95% CI 2.5–110.2)	>> IFX
Cañete et al. 2020 [129]	IFX or ADA	Retrospective cohort	83 IFX, 96 ADA	NA	98/81	Smo = 49% Str = 44%	Endoscopic improvement in 61%; Endoscopic remission in 42%; Thiopurines + IFX >> ADA	Effective
Tursi et al. (2021) [131]	UST	Retrospective case-series	15 clinical and endoscopic cohort	42 (37–52)	9/6	Smo = 13%	Endoscopic remission (11/11); Clinical remission 6 months (12/15)	Uncertain
Macaluso et al. (2023) [132]	UST	Retrospective cohort	44 endoscopic POR	47.3 (±15.0)	18/26	Smo = 42% Str = 84% nR = 6.8%	Endoscopic improvement in 50%; Endoscopic remission in 27%; No endoscopic response in 27%	Uncertain
Macaluso et al. (2022) [134]	VDZ	Retrospective cohort	58 endoscopic POR	36 (18–73)	158/139	nR = 42% Str = 57%	Endoscopic improvement 16 months in 48%; clinical failure 1 year in 19%; new surgery in 12%	Uncertain

M = male; F = female; MTX= methotrexate; RCT = randomized controlled trial; 5-ASA = 5-aminosalicylic acid; AZA = azathioprine; Smo = smokers; nR = number of resections > 1; Str = stricutring phenotype; POR = postoperative recurrence; IFX = infliximab; ADA = adalimumab; UST = ustekinumab; VDZ = vedolizumab; OR = odds ratio.

### 6.8. Non-Pharmacological Treatment

There is no current evidence to suggest the use of prebiotics or probiotics in the treatment of POR [135]. In addition, specific postoperative dietary regimens are yet to be examined.

## 7. Discussion

The management of POR in CD is complex and challenging. Although significant progress has been made in understanding and addressing this disease setting, there are still several crucial gaps that need to be addressed. 

Considering the complexity of CD and the individual variations in patient characteristics and disease behavior, a multidisciplinary approach is essential to ensure a comprehensive assessment [136,137]. The team should consist of gastroenterologists, surgeons, radiologists, and rheumatologists and dermatologists, when necessary, who collaboratively assess the patient’s risk profile and determine the most appropriate treatment. In addition, the involvement of a stoma therapist, navigator nurse, nutritionist, psychologist, and even clinical pharmacist is paramount in the comprehensive management of patients, providing support and guidance throughout the treatment process [138,139,140,141,142]. By involving a multidisciplinary team, the management of POR in CD can be tailored to the specific needs of each patient, taking into consideration risk factors and individualized therapeutic strategies. This approach optimizes the chances of achieving favorable outcomes and minimizing the risk of recurrence.

A substantial obstacle in the management of POR is the absence of consensus on standardized therapeutic algorithms, surveillance frequencies, and treatment strategies. This evidence gap impedes the establishment of optimal strategies, including systematic prophylaxis, risk stratification, and endoscopy-driven interventions. Another significant challenge in managing POR in CD lies in the widespread implementation of reliable tools that can guide clinical decision-making and therapeutic selection. However, FC and IUS hold promise as they can indicate inflammation. Although combining these markers presents an opportunity based on existing research, further validation is necessary. Moreover, there should be increased recognition and utilization of IUS in clinical practice guidelines alongside FC testing. By integrating these non-invasive markers more extensively into management protocols, it is possible to overcome current limitations and pave the way for personalized treatment approaches that optimize POR outcomes through stratified risk assessment.

Moreover, the issue of overtreatment and unnecessary side effects resulting from immediate prophylactic therapy necessitates a more nuanced approach that involves shared decision-making and tailored treatment plans. Factors such as recently active perianal fistulizing disease, concomitant immune-mediated inflammatory diseases, or the occurrence of extra-intestinal manifestations and prior involvement of the colon should be carefully considered. Additionally, active smoking, penetrating disease as an indication for index surgery, and previous intestinal resections may qualify patients for immediate prophylactic therapy, configuring a picture of difficult-to-treat disease [143]. 

The role of immunosuppressive agents and biological therapies in preventing POR requires further investigation, particularly in terms of comparative effectiveness and long-term outcomes. While biologics demonstrate effectiveness, they exhibit high rates of both primary non-response, ranging from 20–40% within the first year of anti-TNF therapy, and secondary loss of response over time [34,144]. Another limitation is the inability of current therapies to reverse established fibrosis [145,146]. Ongoing trials will address the evidence gaps; for instance, the Soprano-CD trial (ClinicalTrials.gov Identifier: NCT05169593) intends to evaluate if endoscopy-driven vs. systematic prophylactic biological therapy leads to more endoscopic POR after 86 weeks. Furthermore, the differential treatment of patients with various endoscopic findings and the natural course of different types of lesions—such as modified RS i1, i2a, and i2b—necessitate more comprehensive research. 

Furthermore, the importance of regular postoperative endoscopic evaluation cannot be overstated, as it provides crucial information for the timely initiation or optimization of prophylactic therapy. Performing the first postoperative endoscopy at six months allows for the identification of early lesions and facilitates treatment adjustments. However, the optimal management approach for specific endoscopic findings remains uncertain, and further research is needed. Despite the clear benefit, ensuring adherence to the recommended endoscopic surveillance can be challenging. Excessive wait times for endoscopic procedures, as well as loss to follow-up of patients, can result in missed opportunities for early intervention. Health systems must prioritize sufficient capacity and work to minimize barriers leading to patient attrition. Solutions such as patient navigators, reminders, and scheduling support are critical to helping patients complete advised postoperative endoscopic surveillance [147].

**Figure 2:**
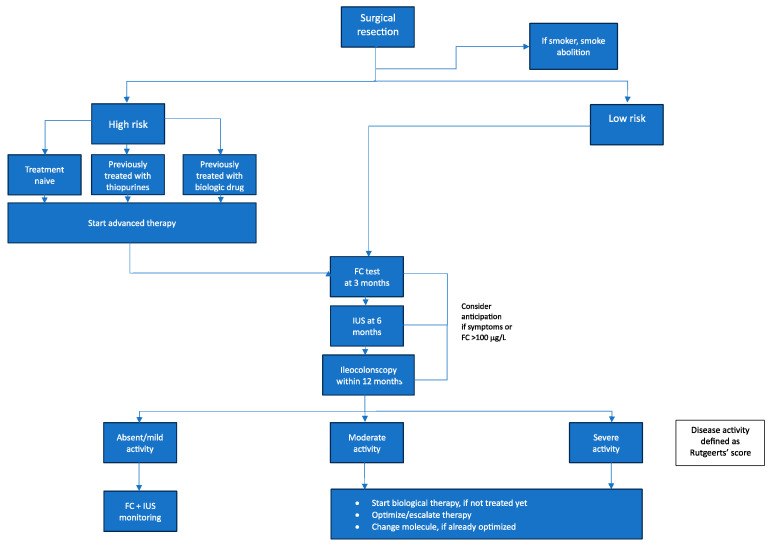
Proposed updated algorithm for the management of postoperative Crohn’s disease. FC = Fecal calprotectin; IUS = intestinal ultrasound.

Based on the current literature, we propose the following treatment algorithm for postoperative patients with CD (Figure 2). This algorithm aims to stratify these individuals according to their risk of POR. Specifically, patients are categorized as either high or low risk for POR. By systematically incorporating risk factors for POR and treatment history, it seeks to provide clinicians with optimized guidance on managing these postoperative CD patients going forward. 

In summary, while progress has been made in managing POR in CD, several challenges persist. Prospective studies addressing the identification of accurate predictors of POR, direct comparisons between postoperative strategies, and the development of personalized treatment algorithms are warranted to improve patient outcomes in this challenging clinical scenario. With ongoing research efforts and the implementation of a multidisciplinary approach involving shared decision-making, clinicians can strive to optimize postoperative strategies and provide tailored therapies.

## 8. Conclusions

POR remains one of the most challenging aspects in the management of CD. Despite advances in medical therapies, many patients still require surgery for complications or refractory disease. However, recurrence is common following resection, with endoscopic lesions identified in up to 80% of patients within one year. Preventing and promptly treating POR is crucial to avoid repeat surgeries and bowel damage. Risk stratification guides treatment approaches, with factors like smoking, penetrating disease, prior resection, and granulomatous inflammation warranting more aggressive prevention in high-risk patients. Combining non-invasive tools, like FC and imaging, provides comprehensive disease assessment to detect early recurrence. Biologics are effective in reducing endoscopic and clinical recurrence, but comparative studies are still lacking. Anti-TNF agents appear efficacious, but other biological drugs and small molecules show promise and require further research [148,149]. While some patients may achieve prolonged remission after surgery, many require lifelong maintenance therapy. However, endoscopy-guided treatment may be reasonable for low-risk patients to limit medication side effects. Ongoing studies are exploring strategies like prophylactic drug withdrawal and comparing systematic prophylaxis to selective endoscopic-driven treatment. Overall, a multidisciplinary approach considering patient preferences and optimizing medication access and adherence is essential. Further studies are needed to provide evidence-based treatment algorithms and validate tools to risk-stratify patients. Advances in medical and preventative strategies provide an opportunity to significantly improve postoperative outcomes in CD.

## Data Availability

Not applicable.
